# Salivary duct carcinoma of the parotid gland: A case report and review of the literature

**DOI:** 10.3892/ol.2014.2655

**Published:** 2014-10-31

**Authors:** SHULE XIE, HONGYU YANG, MARIUS BREDELL, SHIYUE SHEN, HUIJUN YANG, LONG JIN, SHANSHAN ZHANG

**Affiliations:** 1 Department of Oral and Maxillofacial Surgery, Peking University Shenzhen Hospital, Shenzhen, Guangdong 518001, P.R. China; 2 Medical College of Shantou University, Shantou, Guangdong 515041, P.R. China; 3 Department of Cranio-maxillofacial and Oral Surgery, University Hospital Zürich, Zurich 8091, Switzerland

**Keywords:** salivary duct carcinoma, salivary gland, clinic, immunohistology

## Abstract

Salivary duct carcinoma (SDC) is a rare and aggressive parotid malignancy that most commonly affects males in the fifth and sixth decades of life. Histopathology specimens obtained from SDC patients demonstrate a resemblance to ductal carcinoma of the breast. Therefore, to distinguish SDC from breast ductal carcinoma, several immunohistochemical markers exist that may enable surgeons to make an accurate diagnosis. In this study, the case of a 54-year-old male with salivary duct carcinoma of the right parotid gland is presented. The results of the present case study revealed that the SDC sample was positive for the expression of human epidermal growth factor 2 (Her-2), cytokeratin (CK) 8/CK 18, p63, high molecular weight CK and calponin, and negative for expression of the estrogen receptor and progesterone receptor. Based on the result, an ipsilateral selective neck dissection followed by adjuvant post-operative radiation therapy was suitable at the primary treatment stage. At one year of follow-up, the patient was alive and free of recurrence. In advanced cases of SDC, treatment with anti-HER-2 monoclonal antibodies, such as trastuzumab, is recommended.

## Introduction

Salivary duct carcinomas (SDC) are aggressive, high-grade salivary malignancies first described by Kleinsasser *et a*l (1). The tumors are characterized by a histological resemblance to ductal carcinoma of the breast. The reported incidence of SDC is 1–3% among all salivary tumors(2). This tumor exhibits aggressive clinical behavior with a tendency for early cervical lymphadenopathies and distant metastases to the lungs and bones and thus, the prognosis of SDC is highly unfavourable (3). Surgical resection followed by radiation is the treatment of choice, however, locoregional recurrences and distant metastases have frequently been reported (4). The disease is rarely found in the parotid gland. The present case study reviews the clinical data of a patient with SDC in the deep lobe of the parotid gland and discusses the relevant literature. Written informed consent was obtained from the patient.

## Case report

**Figure 1:**
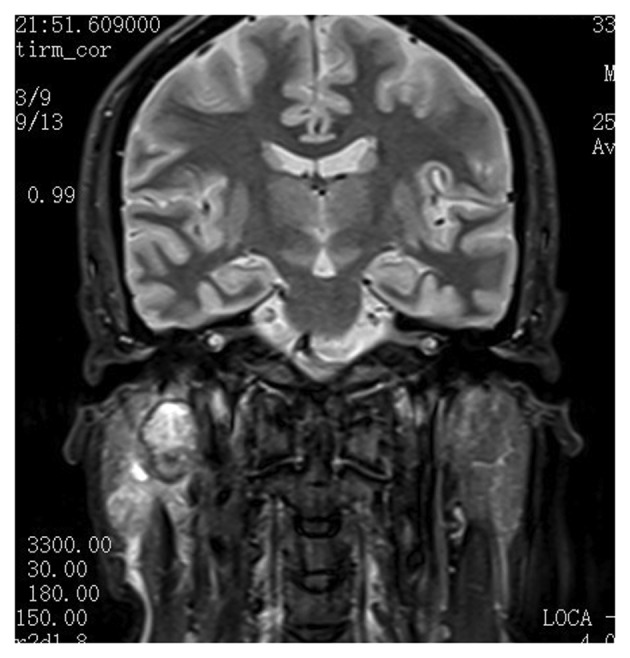
T1-weighted gadolinium enhanced magnetic resonance imaging revealing a tumor signal in the deep lobe of the parotid gland.

A 54-year-old male presented with a moderate, painless swelling of the right parotid region that had been apparent for two weeks. The patient had no history of fever or other constitutional symptoms. A physical examination revealed a firm, but mobile, lump that was not fixed to the overlying skin. The functioning of the facial nerve was within normal limits. Upon clinical examination, palpation identified no enlarged or pathological lymph nodes. Magnetic resonance imaging (Fig. 1) identified a neoplasm of ~2 cm in diameter located in the deep lobe of the parotid gland and involving the exofacial parotid gland. This lesion was clinically and radiologically classified as cT3cNxcM0 according to the World Health Organization International Classification of Tumors (5). Computed tomography of the chest appeared negative for distant metastatic lesions.

**Figure 2:**
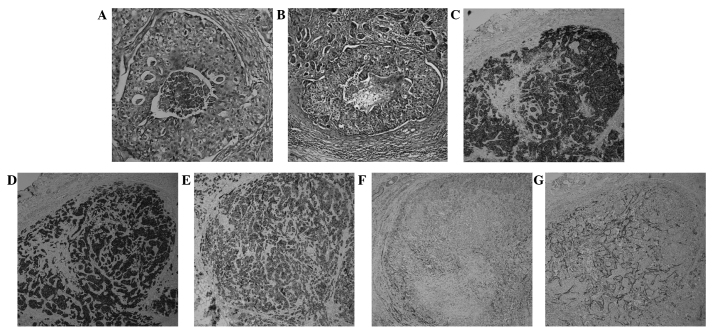
(A) Tumor in the primary site arranged in a glandular pattern with necrosis in the center of the duct (H&E; magnification, ×100). (B) Tumor in the lymph node demonstrating a similar structure as observed in (A) (H&E; magnification, ×100). (C) Positive HER-2 expression in the tumor sample (ABC; magnification, ×100). (D) Diffusely positive CK-H expression and (E) moderately positive CK8/CK18 expression (ABC; magnification, ×100). (F and G) The expression of p63 and calponin located in the myoepithelial surrounding the salivary ducts (ABC; magnification X100). H&E, hematoxylin and eosin; ABC, avidin-biotin complex; CK, cytokeratin; CK-H, high molecular weight CK.

The primary surgical treatment for the patient consisted of a total parotidectomy conserving the facial nerve and a modified ipsilateral radical neck dissection, as the intraoperative histology suggested a malignant tumor. The excision site was covered by a sternocleidomastoid muscle flap. Intraoperatively, surgeons identified eight hard and enlarged lymph nodes at cervical levels III and IV on the right side of the neck. The largest lymph node, which measured 1.5×1.5 cm, was situated at level III and showed early infiltration of the sternocleidomastoid muscle. Frozen sections of the lymph nodes confirmed that six out of eight nodes contained malignant cells, highly suggestive of salivary gland carcinoma (Fig. 2B). Upon final histopathological examination, a diagnosis of SDC was confirmed. All the resection margins were free from tumor and the tumor-free margin was <1.0 cm. In order to further investigate the tissue samples, several immunohistochemical markers were analyzed, including human epidermal growth factor 2 (HER-2), high molecular weight cytokeratin (CK-H), CK8/CK18, p63, calponin, the estrogen receptor (ER) and the progesterone receptor (PR).

The patient experienced no complications during post-operative healing. Upon examination, the surgical margins were clear, therefore, no further surgery was required. Fifteen days after surgery, post-operative radiation therapy (60 Gy; 2 Gy, twice a day, five days a week) was applied to the surgical bed and the right neck area due to the aggressive nature of the tumor. The final pathological staging was pT4pN2pM0, and three years after treatment, the patient remains free from tumor recurrence.

## Discussion

SDCs generally affect males in the fifth or sixth decades of life, with the average age of occurrence at 60 years. Valeri *et a*l (2) declared SDC to be a rare form of parotid tumor originating from the major or minor salivary glands and accounting for 0.2–2% of all salivary gland tumors (3). The majority of cases of SDC present as a rapidly enlarging firm mass accompanied by facial paralysis or pain. Cervical adenopathy and lymph node invasion are identified in 35% (4) and 40–80% (6) of SDC patients, respectively. In the present case study, eight hard lymph nodes were observed along the sternocleidomastoid, with six of them demonstrating histopathological involvement.

In 2005, SDC was defined as an independent entity by the World Health Organization, labeling it as ‘an aggressive adenocarcinoma, which resembled high-grade breast ductal carcinoma’. SDC was previously divided into two categories; low-grade and high-grade SDC. The low-grade SDC was recognized as a rare, cystic, proliferative carcinoma that resembled the spectrum of breast lesions, including atypical ductal hyperplasia and micropapillary and cribriform low-grade ductal carcinoma *in situ* (7). Low-grade SDC has subsequently been defined as a classification termed low-grade cribriform cystadenocarcinoma. Under the current definition of SDC, the present case study defines high-grade SDCs as tumors that consist of solid invasive cancer nests with polygonal cancer cells surrounding a comedo-like necrosis. In the present case study, it was observed that the intraductal component of the primary foci and the malignant lymph nodes exhibited central comedo necrosis associated with a cribriform, solid or micropapillary architecture (Fig. 2A and B). SDC is generally a hematoxylin and eosin stain-based diagnosis, however, specific immunohistochemical and staining techniques may confirm a diagnosis in certain cases, and immunomarkers may be beneficial for future therapeutic approaches. Immunohistochemically, SDC is positive for the expression of low molecular weight CKs and epithelial membrane antigen (8). Nikitakis *et al* (9) demonstrated that CK7 was diffusely positive in the majority of malignant salivary gland tumors and that CK20 was intermittently focally stained. In the present case study, immunohistochemistry of the tumor sample identified that CK-H expression was diffusely positive, whilst CK8/CK18 expression was moderately positive (Fig. 2D and E). SDC lesions are usually negative when stained for the expression of S-100 protein or basal-myoepithelial markers, such as CK 5/6 and 14, p63, calponin and smooth muscle myosin heavy chain (8). However, the present case study revealed that p63 and calponin were weakly positive in the myoepithelium surrounding the ducts, which suggested that the surrounding cells of the *in situ* lesions were neoplastic (Fig. 2F and G). The overexpression of HER2 protein, identified in ~90% of SDC cases (10), was apparent in the present case study (Fig. 2C). Significant differences have been identified between the hormone receptor profiles of SDC and invasive ductal carcinoma of the breast. The presence of the ER and PR is found in 75% of cases of breast cancer, however, positivity for these markers is rare in SDC (9). However, SDC analysis in the present study found the samples to be ER- and PR-negative. Based on these data, Simpson proposed that SDCs could be classified into three main groups: Luminal androgen receptor-positive, HER2-positive and basal phenotype, which may form the basis for prognostic information and novel therapeutic possibilities (8).

Due to the infiltrative nature of SDC, radical surgery is the primary treatment; this involves the surgical removal of the tumor by parotidectomy with or without conservation of the facial nerve, followed by neck dissection to allow for ipsilateral lymph node excision. However, the rate of locoregional recurrence is high and the prognosis for survival is poor in the case of insufficient resection margins, particularly in cases with lymph node invasion (6). Lymphatic embolism and perineural, extraparotid and/or lymphatic invasion are further indicators of a poor prognosis. Post-operative radiation therapy is mandatory in advanced cases of SDC, whereas chemoradiotherapy is generally reserved for metastatic forms of the tumor. The prognosis may be improved in tumors measuring <2 cm (6,11), however, the five-year recurrence-free survival rate remains at ~30% (2,12).

Previous studies have demonstrated that HER2 is an effective therapeutic target for patients with HER2-positive breast cancers. di Palma *et al* (1) suggested that certain individuals with advanced SDC treated with trastuzumab (an anti-HER2 monoclonal antibody) demonstrated promising results. Therefore, patients with HER2 subtype SDCs may benefit from targeted therapies using anti-HER2 monoclonal antibodies, including trastuzumab and pertuzumab, or HER2 tyrosine kinase inhibitors, such as lapatinib.

SDC is a rare and aggressive salivary gland malignancy for which treatment is surgical resection and neck dissection, with adjuvant radiation therapy reserved for the more advanced forms. The current report may increase knowledge with regard to SDCs. The primary clinical symptom presented by the patient in this case was a painless mass in the right deep parotid. Therefore, the pathological and immunohistochemical analysis of SDC is required to diagnose patients with a painless mass in the deep parotid, in order to avoid misdiagnosis. Furthermore, since SDC usually develops aggressively with the possibility of early distant metastasis and local recurrence, this indicates that surgery and postoperative radiation are beneficial for SDC patients. HER2-targeted therapies may therefore be a novel and effective future treatment choice for certain SDC patients. Furthermore, additional studies focusing on the etiology and mechanism of SDC are required.

## Abbreviations

SDC: salivary duct carcinoma
HER2: human epidermal growth factor receptor-2
ER: estrogen receptor
PR: progesterone receptor
CK: cytokeratin
